# Reading Instructions Influence Cognitive Processes of Illustrated Text Reading Not Subject Perception: An Eye-Tracking Study

**DOI:** 10.3389/fpsyg.2018.02263

**Published:** 2018-11-29

**Authors:** Yu-Cin Jian

**Affiliations:** ^1^Department of Educational Psychology and Counseling, National Taiwan Normal University, Taipei, Taiwan; ^2^Institute for Research Excellence in Learning Science, National Taiwan Normal University, Taipei, Taiwan

**Keywords:** eye movements, reading instructions, illustrated text, cognitive processes, subjective perception

## Abstract

Scientific illustrations play an important role in scientific texts, however, young readers show limited ability to use illustration information and integrate it with the text in multimedia learning. The primary goal of the present study was to investigate if strategy instructions for illustrated text reading focused on scientific illustration reading and text-illustration integration can help young readers overcome their deficiencies and change their reading processes, learning outcomes, and subjective perceptions of article difficulty and enjoyment, illustration enjoyment, and self-evaluation of learning. Besides, is subjective perception of the article related to reading behavior? Sixty-two fourth-grade students read an illustrated science text while their eye movements were recorded, and then completed a reading test and questionnaire. The instruction group outperformed the control group on the reading test, but subjective perceptions of the article did not differ between groups. Eye-movements analysis showed that the instruction group spent twice as much time reading the illustrations and made more saccades between relevant text and illustration sections than the control group. These findings indicate that strategy instructions for reading illustrated text promoted reading comprehension and changed learning processes, not subjective perceptions. In addition, the result of this study showed that there was no relationship between subjective perception (article difficulty or illustrations enjoyment) of the article and reading time (total reading time on the illustrated science text and on the science illustrations). This study had empirical and practical contributions. Empirically, this study developed the instruction methods in multimedia learning and further examined their effect on learning processes in young readers. Practically, this study can help elementary school teachers understand the processes used by young readers when reading illustrated texts and provide them with evidence-based instructions to teach science reading effectively.

## Introduction

Scientific diagrams play an important role in scientific texts (Pozzer and Roth, 2003; Slough et al., 2010): they can provide a concrete example of an abstract concept in the text description, and often provide additional information that cannot be easily provided in the text description, such as part-whole relationships in an organism. Several learning theories about illustrated text reading, such as the cognitive theory of multimedia learning (Mayer, 2014) and integrated model of text and picture comprehension (Schnotz and Bannert, 2003; Schnotz et al., 2014), also indicate the importance of illustrations in multimedia learning. However, previous research clearly indicates that young readers in elementary school pay little attention to scientific diagram information and do not use it during scientific text reading (Moore and Scevak, 1997; Hannus and Hyönä, 1999; McTigue, 2009; Norman, 2012; Jian, 2016). Jian (2018) found that teaching fourth-grade students strategies for reading illustrated text promoted reading comprehension, and, importantly, the facilitation effect was found in students with both low and high reading ability. This study further investigated this facilitation effect using eye-tracking technology.

### Young Readers’ Strategies for Reading Illustrated Scientific Texts

Think-aloud protocols and eye-tracking technology are two research methods that are often used to investigate young readers’ reading strategies and reading processes for illustrated scientific texts. In think-aloud protocols, participants say whatever comes into their mind (e.g., what they are looking at, thinking, doing) as they are reading articles. This makes thinking processes explicit during reading (Ericsson and Simon, 1984; Moore and Scevak, 1997). Eye-tracking technology yields on-line recordings of where learners are looking from moment to moment, and how long they fixate on different segments of the learning material, such as text or illustrations. There is a close relationship between gaze location and attention engagement during reading (Rayner et al., 2006; van Gog and Scheiter, 2010). According to the eye-mind assumption (Just and Carpenter, 1980), the distribution of visual attention indicates what is being processed and for how long. This is the basic idea behind eye-movement research on reading.

Moore and Scevak (1997) used think-aloud protocols to investigate developmental differences in reading strategies during illustrated text reading. They asked fifth-, seventh-, and ninth-grade students to read an illustrated science text, and analyzed their thing-aloud data. Compared to younger students, older students used more diverse reading strategies and explicitly linked text and illustration information. The percentages of students that used illustration information were 8, 13, and 48% for fifth-, seventh-, and ninth-grade students, respectively. The younger students made more verbal reports on text details, but rarely thought the illustration information helped them understand the text information. Using the same think-aloud protocol, Norman (2012) investigated the relationship between children’s reading processes prompted by graphics in informational texts and reading comprehension. The two texts were about dinosaurs and weather. Second-grade students shared their thoughts as they read each graphic, then retold the article content in their own words and completed a comprehension test. There was a positive correlation between graphic use and retelling, but not comprehension, measures. It appears that elementary school students have limited ability to use illustration information to help them understand article content.

Using eye-tracking technology, Hannus and Hyönä (1999) investigated young readers’ attentional guidance during illustrated text reading. Fourth-grade students with high and low intelligence were asked to read illustrated science passages. High-intelligence students were more strategic and spent more time on relevant text and illustration sections. However, both groups spent surprisingly little time (approximately 6%) looking at the illustrations. The researchers concluded that reading was heavily text-driven for young readers. In my recent study, I (Jian, 2016) also used eye-tracking technology to investigate differences in reading processes between adult and young readers. Undergraduate students and high-ability fourth-grade students were asked to read an illustrated science article, and their eye movements were recorded. Adult readers, but not young readers, made frequent transitions between relevant text and illustration sections. Moreover, the percentage of total reading time spent on illustrations in the learning episode was 26% for adult readers, but only 18% for young readers.

In sum, studies on illustrated text reading in young readers suggest that they devote little attention to illustrations and have a limited ability to use text-illustration reading strategies during illustrated text reading. The cognitive theory of multimedia learning (Mayer, 2014) and integrated model of text and picture comprehension (Schnotz and Bannert, 2003; Schnotz et al., 2014) have proposed using multiple textual and pictorial representations to help readers memorize and comprehend better.

### Ways to Foster Text-Illustration Integration

Designing signals on reading materials (e.g., using labels and highlighting as visual cues) (Mautone and Mayer, 2001; Scheiter and Eitel, 2015) and teaching (e.g., eye movement modeling examples, reading strategy instructions) (van Gog et al., 2009; Mason et al., 2015) are often used to foster text-illustration integration in students or novices. Both methods are based on the theoretical framework of multimedia learning: the cognitive theory of multimedia learning (Mayer, 2014) and integrated model of text and picture comprehension (Schnotz and Bannert, 2003; Schnotz et al., 2014) propose using textual and pictorial information to helps readers link multiple representations into a mental model, and thus improve learning outcomes.

Scheiter and Eitel (2015) used an eye-tracker to investigate the effects of signaling on visual attention and learning outcomes. They asked undergraduate students to read a 16-page article about the structure and functioning of the human circulatory system with or without signals on the article. The signals group outperformed the no-signals group on a subsequent reading test. Furthermore, the signals group attended more frequently and earlier to signaled elements in the illustration than the no-signals group, whereas both groups attended equally often to non-signaled elements in the illustration. These results suggest that corresponding signals guided attention toward signaled elements and fostered learning outcomes. Other research also found that readers spent more time on signaled elements in reading materials (e.g., Kriz and Hegarty, 2007; Boucheix and Lowe, 2010; de Koning et al., 2010); however, de Koning et al. (2010) found that signaling had no impact on learning outcomes.

An alternative method is eye-movement modeling example (EMME), in which eye tracking is used to support learners’ orientation of attention in video-based modeling examples (Jarodzka et al., 2013), which can focus cognitive resources on the correct solution or way to perform a task (Mason et al., 2015). Mason et al. (2015) tested whether replaying a model’s eye movements while reading an illustrated text would benefit readers’ text-diagram integration behavior and promote reading comprehension. Forty-two seventh-grade students were randomly assigned to an experimental (EMME) or control (No-EMME) group. The experimental group watched a short video showing the eye movement replay of a model reading an illustrated text about the water cycle, but without simultaneous verbal explanations. The model behaved didactically to deliberately model an integrative reading strategy. The control group did not watch this video, but read the illustrated text by themselves. Then, both groups read another illustrated text about the food chain while their eye movements were recorded. Students in the EMME condition performed better on the reading test than students in the No-EMME condition. The students in the EMME condition also spent more rereading time on corresponding text and illustration sections. van Gog et al. (2009) also used eye-movement modeling examples as an instructional strategy, and replayed recorded eye positions of a skillful expert to less skillful students with the aim of helping them acquire the desired skills.

The findings described above indicate that signals and eye-movement modeling examples are effective ways to foster text-illustration integration when reading science articles. However, these studies used middle-school or university students as participants, so the conclusion might not generalize to the younger fourth-grade students of interest in this study. Fourth-grade students are at the beginning stage of read to learn (Chall, 1983), so their diagram literacy might not be mature enough to encode illustration information (Kress and van Leeuwen, 1996; Moore and Scevak, 1997). Therefore, during eye-movement modeling examples, young readers may follow and look at the model’s eye fixations, but may not know what information needs to be extracted from the illustrations; seeing does not necessarily equal understanding (de Koning et al., 2010). A similar concern applies to using signals: young readers may see colors or labels but this does not guarantee that they understand their conveyed meaning. Furthermore, following a model’s eye movements and simultaneously understanding what the model is thinking is cognitively demanding. Young readers have limited cognitive resources, so cognitive overload may result in poor comprehension performance.

The third possible method for fostering text-illustration integration in young readers is reading instructions. Little research to date has investigated multimedia strategy instructions and learning outcomes, and the results are inconclusive. In a recent research, I (Jian, 2018) designed an instruction experiment to teach illustration reading and text-illustration integration strategies to fourth-grade students, who then read illustrated science texts. High- and low-ability students in the instruction group outperformed students in the control group on the reading test. It was encouraging to discover that teaching students to pay attention to illustrations, process them in-depth, and consider the relationship between textual descriptions and detailed parts of illustrations led to benefits in learning scientific knowledge even in low-ability students. Scheiter et al. (2015) also developed a multimedia strategy training for ninth-grade students. They found that although students could learn reading strategies, they did not apply them to new reading situations.

### Experiment Overview and Hypotheses

The primary goal of the present study was to investigate if strategy instructions for illustrated text reading focused on scientific illustration reading and text-illustration integration can help young readers overcome their deficiencies and change their reading processes, learning outcomes, and subjective perceptions of article difficulty and enjoyment, illustration enjoyment, and self-evaluation of learning.

Students in the experimental group received brief reading instructions, then they read formal learning material (an illustrated science text) independently and completed a reading test that included memorization, comprehension, and text and illustration integration questions. Finally, they completed a questionnaire about subjective perception of the reading material. The procedure was the same for students in the control group, with the exception that they did not receive the reading instructions. Reading ability, prior knowledge about the reading material, and reading self-efficacy did not differ between groups (see “Methods” section). The research questions and hypotheses of this study are as follows:

(1) Do reading strategy instructions promote reading performance?
  According to the cognitive theory of multimedia learning (Mayer, 2014) and integrated model of text and picture comprehension (Schnotz and Bannert, 2003; Schnotz et al., 2014) based on dual-coding theory (Paivio, 1990), readers who use textual and pictorial representations and integrate them outperform readers who only use single representations to construct mental models of leaning episodes. Therefore, students in the instruction group were expected to show better learning outcomes than students in the control group (Hypothesis 1a). Due to the content of the reading instructions, the strongest effects were expected for text-illustration integration and illustration memorization questions (Hypothesis 1b).
(2) Do reading strategy instructions change learning processes?
  Previous research (Hannus and Hyönä, 1999; Jian and Ko, 2017) has shown that young readers with different levels of reading ability engage different learning processes during illustrated text reading. Therefore, if reading strategy instructions promote reading ability, then students in the instruction and control groups should express different reading processes. Because the instructions focused on illustration reading and text-illustration integration, students in the instruction group should devote more cognitive effort to reading the illustration section (i.e., more total reading time and more fixations on illustrations; Hypothesis 2a). In particular, they should show more transitions (saccades) between text and illustrations (Hypothesis 2b), especially for related text and illustration sections (i.e., more saccades from paragraph 2 to the top illustration, and from paragraph 3 to the bottom illustration; Hypothesis 2c). Moreover, students in the instruction group were expected to attend to illustrations (greater proportion of total reading time on illustrations, Hypothesis 2d) in a similar manner as mature (adult) readers, who spend approximately 20–30% of reading time on illustrations during science reading (Jian and Wu, 2015; Jian, 2016).
(3) Do reading strategy instructions influence subjective perceptions of article difficulty, article enjoyment, illustration enjoyment, and self-evaluation of learning?
  Previous research fostering text-illustration integration does not speak to this question (Mautone and Mayer, 2001; van Gog et al., 2009; Mason et al., 2013b, 2015; Scheiter and Eitel, 2015), because they only measured cognitive comprehension, not reading attitudes. On the one hand, subjective perceptions may not change in such a short time, as the reading instructions only took about 10–20 min (see “Method” section). Therefore, I hypothesize that there should be no significant difference between the groups with and without reading instructions in terms of questionnaire ratings of article/illustration likeness (Hypothesis 3a). On the other hand, learning reading strategies might increase self-evaluations of learning performance (Hypothesis 3b).
(4) Is subjective perception of the article related to reading behavior? For example, do readers who feel the article is more difficult spend more time reading or give up more quickly? Do readers who feel that the illustration was more attractive spend more time looking at the illustration?
In general, more time is needed to understand difficult texts. However, if readers feel a text is very difficult, they may give up and terminate reading as quickly as possible. Some previous research consistent with this possibility showed that high-ability students spent more time reading articles than low-ability students (Hannus and Hyönä, 1999; Jian and Ko, 2017). Therefore, I have no hypothesis about the relationship between subjective article difficulty and reading time (Hypothesis 4a). According to the theory of motivation, which indicates that interest is related to engagement (Ainley, 2006; Unrau and Quirk, 2014), illustration enjoyment was expected to be positively related to illustration reading time (Hypothesis 4b).

## Methods

### Participants

Initially, 76 fourth-grade students were recruited from four classes at two elementary school to complete a standard reading comprehension screening test (Ko, 2006). The consent obtained from the parents of all research participants was both informed and written. Ten students with accuracy below 30% were excluded because they may have reading difficulties. Thus, 66 students participated in the experiment. Half of the students were individually assigned to the instruction group (14 girls and 17 boys; mean age = 10.3 years), and half were assigned to the control group (16 girls and 15 boys; mean age = 10.2 years). Participants’ reading ability was measured by a standard reading comprehension screening test (Ko, 2006), and it did not differ between groups (*p* > 0.05). The average *Z*-score of the standard reading test was 0.51 (range = -0.68 to 1.30, *SD* = 0.61) in the instruction group, and 0.57 (range = -0.65 to 1.40, *SD* = 0.67) in the control group. This study was approved by the research ethics committee (REC) in National Taiwan Normal University, and the REC number was 201512HM010.

### Materials

The materials consisted of three pre-tests, a practice article, a formal article, a posttest, and a questionnaire. The three pre-tests were used to determine whether the groups were equivalent in terms of basic characteristics, and included a standard reading comprehension screening test (Ko, 2006), a prior knowledge test, and a reading self-efficacy questionnaire. The standard reading comprehension-screening test consisted of 32 multiple-choice questions that measured literal meaning, alleged pronoun, sentence comprehension, and text comprehension abilities. The prior knowledge test consisted of 12 yes/no questions relevant to the topic of the reading material (flower structure). The reading self-efficacy questionnaire (Mullis et al., 2009) consisted of seven items (please see Appendix A), such as “Reading is easy for me” and “I usually do well in reading.” Students answered each question using a 4-point scale (1 = strongly agree, 4 = strongly disagree).

The learning materials included a practice and formal article. The practice article included a science text about respiration and two illustrations depicting air exchange in earthworms and humans. This practice article was used as an example of reading strategies in the instruction group and to familiarize readers with the form of the formal article. The formal article was the same as used in the research (Jian, 2016) modified from a science textbook (Huang, 2013). It introduced the forms and functions of a flower and consisted 400 Chinese words and two illustrations. The text was divided into three paragraphs that introduced plant reproduction, a flower’s structure and functions, and pollination by bees. One illustration was representational, it depicted the detailed structure of a flower with labels, and the other was decorative, it depicted a bee gathering flower nectar. Each of the learning materials was displayed on a single screen, and there was no scroll bar or additional pages. The text section was on the left, and the illustration section was on the right. This arrangement was the same as previous research on illustrated text reading (Mason et al., 2013a; Jian and Wu, 2015; Scheiter and Eitel, 2015; Jian, 2016; Jian and Ko, 2017).

The posttest assessed learning using recognition and comprehension questions (Appendix B). This reading test was used in my previous study (Jian, 2016). *Recognition questions* consisted of 14 multiple-choice questions, half of the questions measured text memory and half measured illustration memory. *Comprehension questions* consisted of 15 yes/no questions, three of which measured text comprehension, three measured illustration comprehension, and three measured text-illustration integration. Three experts assessed the difficulty and readability of the learning materials and comprehension posttest, including a professor in reading psychology, a Ph.D. candidate in science education, and an elementary school science teacher (with a master’s degree in education).

The questionnaire consisted of four items (article difficulty, text enjoyment, illustration enjoyment, and self-evaluation of learning) to understand participants’ subjective perception of the reading material using a 5-point response scale (1 = very easy/like/good, 5 = very difficult/dislike/poor). Appendix A provides the questions in the subjective perception questionnaire.

### Apparatus

Eye movements were recorded using an Eyelink 1000 at a sampling rate of 1000 Hz integrated with a 24-inch LCD monitor with a resolution of 1920 pixels × 1200 pixels. A chin bar was used to minimize head movement. The distance between the monitor and participants was 65 cm. Thus, the learning material covered 46° (horizontal) × 30° (vertical) of visual angle on the screen.

### Procedure

Students were first required to fill in paper-pencil versions of the standard reading comprehension-screening test (Ko, 2006) in their classroom. One week later, students that met reading standards (reading test accuracy higher than 30%) participated in the eye-movement experiment. Students were randomly assigned to the instruction or control group. Both groups had the same number of participants and equivalent average reading ability. Then, participants completed the reading self-efficacy questionnaire and the prior-knowledge test on the computer, followed by the eye-movement experiment.

In the experiment, the instruction group was first taught three strategies (the same as Jian, 2018) for reading illustrated texts. The instructions were as follows: “I will teach you three reading strategies to help you read articles better. The first strategy is to pay attention to the sentences that are relevant to the illustrations. Read these sentences carefully and observe whether the characteristics of the illustration are identical to those in the descriptive sentences. The second strategy is to read every label on the illustrations and carefully study the characteristics of all objects indicated by the labels, for example, shapes, sizes, relative positions, and relationships between components. The third strategy is to read illustration titles, and worded explanations of the illustrations, and observe whether the illustration characteristics are consistent with the information provided in the words on the illustrations.” An assistant researcher taught the participants each reading strategy followed by an example practice article (in paper form) to illustrate how to use the strategy during reading. After the demonstration with the practice article, participants were asked to recall the three reading strategies and indicate how to use them to read the same practice article. If participants omitted a reading strategy or gave an unclear description, the assistant researcher taught him/her again. Then, participants were asked to use the three strategies that they learned to read the same practice article again on the monitor with eye-tracking equipment and answer two questions. After verifying that they were indeed capable of using these strategies to read the practice article, the formal eye-movement experiment began.

Thirteen-point calibration and validation of eye movements was conducted for each participant. Participants were instructed to keep their heads still while reading. Participants were instructed to use the three reading strategies to read the illustrated text for comprehension. Then, they completed a reading test. Finally, participants completed a questionnaire on their subjective perception of article difficulty, article enjoyment, illustration enjoyment, and self-evaluation of learning. The procedure was identical for the control group with the exception that they did not receive the reading instructions. The experiment lasted approximately 30–40 min.

### Data Scoring

#### Eye Movements

Eye-movement data from four participants were discarded due to unsuccessful eye-tracker recordings (two participants) or apparent drift (two participants). Thus, data from 62 participants (31 in the instruction group and 31 in the control group) were analyzed.

Several eye-movement indicators (Hannus and Hyönä, 1999; Kriz and Hegarty, 2007; Boucheix and Lowe, 2010; de Koning et al., 2010; Mason et al., 2013b, 2015; Jian et al., 2014; Jian and Wu, 2015; Jian, 2016) were included in the analyses. (1) Total reading time (the sum of all fixation durations in an area of interest [AOI]), which provides an indicator of the overall difficulty and degree of cognitive effort required to process reading materials. Total reading time of the whole article, text, and illustrations were computed separately. (2) Number of fixations (sum of all fixations in an AOI), which reflects how much attention and cognitive investment readers devoted to the reading material. The number of fixations on text and illustration sections was computed separately. (3) Proportion of total reading time (fixation duration in specific AOIs divided by total fixation duration for the whole article), which reflects selective attention to specific target regions during learning. Proportion of total reading time on text, illustrations, and six AOIs (title, paragraph 1, paragraph 2, paragraph 3, top illustration, and bottom illustration) are reported. (4) Number of saccades between the text and illustrations (the sum total of saccades between text and illustrations), which reflects integration between text and illustration information. The number of saccades from the text to illustrations and vice versa are reported, as well as the number of saccades between relevant text and illustration sections (e.g., paragraph 2 to top illustration, paragraph 3 to bottom illustration).

#### Comprehension Test

The comprehension test included yes/no and multiple-choice questions. Correct answers were awarded one point each, and total scores were converted to percentages.

#### Subjective Perception Questionnaire

Questionnaire items were rated on a 5-point scale. For the article difficulty item, 1 indicated the article was very easy, and 5 indicated the article was very hard. For article and illustration enjoyment items, 1 indicated “like a lot,” and 5 indicated “dislike a lot.” For the self-evaluation of learning item, 1 indicated “I learned all the article content,” and 5 indicated “I learned nothing about the article.”

## Results

Cohen’s *d* is reported as a measure of effect size for *t*-tests. Cohen’s *d* of 0.30, 0.50, and 0.80 are considered small, medium, and large effects, respectively (Cohen, 1988).

### Prior-Knowledge Test and Reading Self-Efficacy Questions

The equivalence of demographic variables between readers in the instruction and control groups was tested first. Means and *SD*s for prior-knowledge test accuracy and reading self-efficacy ratings are presented in Table 1. There were no significant differences between the instruction and control groups in prior-knowledge test accuracy or reading self-efficacy ratings, *p*s > 0.05. Thus, the groups are equivalent in terms of the control variables (prior knowledge and reading self-efficacy).

**Table 1:** Accuracy on the prior-knowledge test and reading efficacy for instruction (*N* = 31) and control (*N* = 31) groups.

	Instruction group		Control group		*t*-value
	*M*	(*SD*)	*M*	(*SD*)	
Prior-knowledge test (6 items)	2.46	(1.08)	2.64	(0.96)	-0.70
Reading self-efficacy (5-point scale)	1.91	0 (0.36)	1.78	0 (0.43)	1.10

### Learning Outcomes

Research question 1 asked whether reading strategy instructions promoted reading performance. This question was answered by examining reading test performance. The results of independent samples *t*-tests are shown in Table 2.

**Table 2:** Accuracy on the reading test for instruction (*N* = 31) and control (*N* = 31) groups.

	Instruction group		Control group		*t*-value
	*M*	(*SD*)	*M*	(*SD*)	
Recognition questions (%)					
Textual items (7)	71	(16)	70	(18)	0.30
Illustration items (7)	78	(14)	65	(24)	2.58^∗^
Comprehension questions (%)					
Textual items (5)	63	00 (22)	59	00 (19)	0.86
Illustration items (5)	66	00 (19)	64	00 (28)	0.32
Integration items (5)	72	00 (21)	50	00 (25)	3.80^∗∗∗^
The whole reading test (29) (%)	71	00 (9)	61	00 (13)	3.50^∗∗^

*^∗^p < 0.05, ^∗∗^p < 0.01, ^∗∗∗^p < 0.001*.

Reading test accuracy was significantly higher for the instruction versus control group (Hypothesis 1a), *t*(60) = 3.50, *p* < 0.01, *d* = 0.94. Furthermore, recognition accuracy was significantly higher for the instruction versus control group for illustration items, *t*(60) = 2.58, *p* < 0.05, *d* = 0.66, but not for text items, *p* > 0.05. Additionally, integration question accuracy was significantly higher for the instruction versus control group, *t*(60) = 3.80, *p* < 0.001, *d* = 1.04. These results are consistent with the hypothesis that the strongest effects would be observed for text-illustration integration and illustration memorization questions (Hypothesis 1b). There was no significant difference in comprehension accuracy for text or illustration items between groups, *p*s > 0.05.

### Eye-Movement Analysis

**FIGURE 1:**
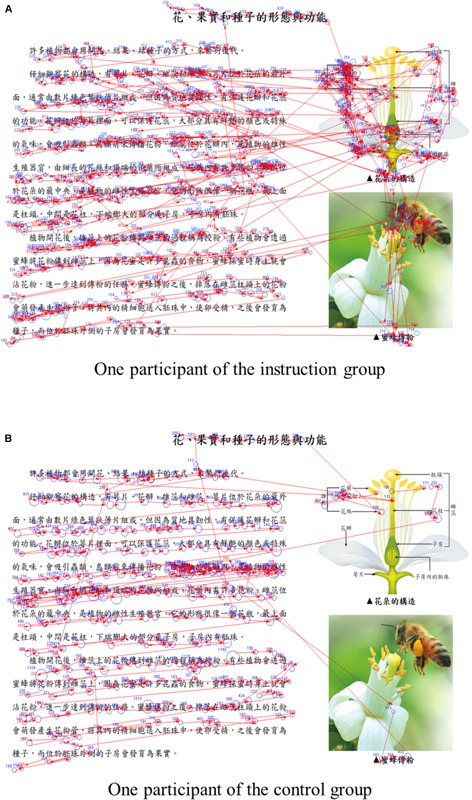
Eye movements of two participants. **(A)** Participant A belonged to the instruction group and **(B)** participant B belonged to the control group.

Research question 2 asked whether reading strategy instructions changed learning processes, including cognitive investment, visual attention distribution between text and illustrations, and reference to text and illustrations, when reading an illustrated science text. This question was answered by analyzing several eye-movement indicators in both groups. Independent samples *t*-tests were conducted on the eye-movement indicators (see below). Figure 1 depicted the eye movement patterns of the two participants in this study, one belonged to the instruction group, and the other belonged to the control group.

#### Analyses of the Whole Article, Text, and Illustration Sections

Total reading time, number of fixations, proportion of total reading time, and number of saccades between text and illustrations were dependent variables in these analyses. Means and *SD*s for these measures are presented in Table 3. Total fixation duration was significantly longer for the instruction versus control group for the whole article, *t*(60) = 2.02, *p* < 0.05, *d* = 0.51, and the illustrations, *t*(60) = 4.74, *p* < 0.001, *d* = 1.20, but not the text, *p* > 0.05. The instruction group made significantly more fixations than the control group on illustrations, *t*(60) = 4.98, *p* < 0.001, *d* = 1.27, but not the text, *p* > 0.05. The instruction group spent a significantly higher proportion of total reading time on illustrations, *t*(60) = 4.34, *p* < 0.001, *d* = 1.08, and a significantly lower proportion of total reading time on the text, *t*(60) = -4.16, *p* < 0.001, *d* = -1.08, than the control group. In addition, the instruction group made significantly more saccades from the text to illustrations and from illustrations to the text than the control group, *t*(60) = 5.14, *p* < 0.001, *d* = 1.30, *t*(60) = 3.17, *p* < 0.01, *d* = 0.67. Furthermore, the instruction group made more saccades between semantically related text and illustration sections (from paragraph 2 to the top illustration, and from paragraph 3 to the bottom illustration) than the control group, *t*(60) = 4.71, *p* < 0.001, *d* = 1.20, *t*(60) = 3.06, *p* < 0.01, *d* = 0.77.

**Table 3:** Means and standard deviations for eye-movement measures for instruction (*N* = 31) and control (*N* = 31) groups.

	Instruction group		Control group		*t*-value
	*M*	(*SD*)	*M*	(*SD*)	
Whole article					
Total reading time (sec)	173.14	(81.09)	138.32	(51.26)	2.02^∗^
Text section					
Total reading time (sec)	123.59	(62.92)	113.94	(49.05)	0.67
The number of fixations	471.16	(226.62)	407.61	(175.43)	1.24
Proportion of total reading time (%)	71	(8)	82	(12)	-4.16^∗∗∗^
Saccades number from text to illustrations	16.48	(10.55)	5.94	(4.39)	5.14^∗∗∗^
Saccades number from paragraph 2 to up illustration	10.06	(7.81)	3.06	(2.74)	4.71^∗∗∗^
Saccades number from paragraph 3 to bottom illustration	2.32	(2.14)	0.94	(1.34)	3.06^∗∗^
Illustration section					
Total reading time (sec)	46.68	(24.83)	21.71	(15.63)	4.74^∗∗∗^
The number of fixations	177.23	(97.22)	77.48	(54.49)	4.98^∗∗∗^
Proportion of total reading time (%)	27	(8)	16	(12)	4.34^∗∗∗^
Saccades number from illustration to text	12.87	(10.63)	7.29	(5.20)	3.17^∗∗^

*^∗^p < 0.05, ^∗∗^p < 0.01, ^∗∗∗^p < 0.001.*

#### Analyses of Specific Articles Areas

To determine readers’ distribution of attention over the reading material, the illustrated text was divided into six AOIs: the title, three paragraphs, and two illustrations, and the proportion of reading time for each AOI was calculated. Means and *SD*s for the proportion of reading time are presented in Table 4. The instruction group spent a significantly greater proportion of reading time on the top and bottom illustrations compared to the control group, *t*(60) = 4.09, *p* < 0.001, *d* = 1.04; *t*(60) = 2.88, *p* < 0.01, *d* = 0.73, while the control group spent a significantly greater proportion of reading time on paragraph 2 compared to the instruction group, *t*(60) = 4.10, *p* < 0.05, *d* = 1.04. Groups did not differ in proportion of total reading time for the title, paragraph 1, and paragraph 3, *p*s > 0.05.

**Table 4:** Means and standard deviations for proportion of total reading time for instruction (*N* = 31) and control (*N* = 31) groups.

	Instruction group		Control group		*t*-value
	*M*	(*SD*)	*M*	(*SD*)	
Title	1%	(1%)	1%	(1%)	0.80
Paragraph 1	4%	(2%)	5%	(3%)	-0.96
Paragraph 2	42%	(9%)	51%	(10%)	-4.10^∗∗∗^
Paragraph 3	24%	(8%)	26%	(9%)	-0.50
Top illustration	23%	(7%)	13%	(11%)	4.09^∗∗∗^
Bottom illustration	4%	(3%)	2%	(2%)	2.88^∗∗^

*^∗∗^p < 0.01, ^∗∗∗^p < 0.001.*

In sum, the results are consistent with Hypotheses 2a–2d. Students in the instruction group devoted more cognitive effort to reading the illustration section (total reading time, number of fixations on illustrations; Hypothesis 2a), and made more transitions between the text and illustrations (number of saccades between text and illustrations; Hypothesis 2b), especially for related text and illustration sections (number of saccades from paragraph 2 to the top illustration, and from paragraph 3 to the bottom illustration; Hypothesis 2c). Moreover, students in the instruction group redistributed their attention from the text to the illustrations (proportion of total reading time on illustrations, Hypothesis 2d).

### Subjective Perceptions Questionnaire

Research question 3 asked whether reading strategy instructions influenced subjective perceptions of article difficulty, article enjoyment, illustration enjoyment, and self-evaluation of learning. This question was answered by analyzing the questionnaire results. Table 5 shows that groups did not differ in the subjective ratings of article difficulty, article enjoyment, illustration enjoyment, and self-evaluation of learning (Hypothesis 3), *p*s > 0.05.

**Table 5:** Questionnaire ratings (5-point scale) of subjective perception of the science article for instruction (*N* = 31) and control (*N* = 31) groups.

	Instruction group		Control group		*t*-value
	*M*	(*SD*)	*M*	(*SD*)	
Article difficulty	3.29	(0.59)	3.52	(1.09)	-1.01
Article likeness	2.10	0 (1.04)	1.84	0 (0.43)	0.98
Illustration likeness	2.23	0 (0.99)	1.94	0 (0.96)	1.17
Self evaluation of learning	2.81	0 (0.75)	2.74	0 (0.89)	0.31

### Relationships Between Questionnaire Ratings, Eye-Movement Indicators, and Reading Performance

Research question 4 asked whether subjective perception of the article was related to reading behavior and reading performance. For example, did readers who felt the article was more difficult spend more time reading or give up more quickly? Did readers who felt the illustrations were more attractive spend more time looking at the illustrations? This question was answered by analyzing correlations between questionnaire ratings, eye-movement indicators (total reading time on the whole article and illustrations), and the reading comprehension test score. Pearson’s *r*-values are reported in Table 6.

**Table 6:** Correlations between questionnaire ratings, eye movements, and reading performance.

Variable	1	2	3	4	5	6	7
(1) Article difficulty		0.27^∗^	0.15	0.24	0.09	-0.09	-0.24
(2) Article likeness			0.42^∗∗^	0.03	-0.04	-0.11	-0.12
(3) Illustration likeness				0.11	0.01	0.00	-0.07
(4) Self evaluation of learning					0.18	0.07	-0.01
(5) Total reading time of the article						0.67^∗∗∗^	-0.11
(6) Total reading time of the illustrations							-0.38^∗∗^
(7) The reading comprehension test score							

*^∗^p < 0.05, ^∗∗^p < 0.01, ^∗∗∗^p < 0.001*.

Because the questionnaire ratings of article difficulty, article enjoyment, illustration enjoyment, and self-evaluation learning did not differ between groups, data for the two groups were pooled in the subsequent analysis. There were significant positive correlations between article difficulty and article enjoyment, *r* = 0.27, *p* < 0.05, article enjoyment and illustration enjoyment, *r* = 0.42, *p* < 0.01, and total article reading time and total illustration reading time, *r* = 0.67, *p* < 0.001. However, there were no significant relationships between subjective article difficulty and reading time (Hypothesis 4a) and illustration enjoyment and illustration reading time (Hypothesis 4b), *p*s > 0.05. In addition, the subjective perception of the article had no significant correlation to the reading test scores, *p*s > 0.05. However, the higher the reading test score, the longer the reading time spent on the illustrations, *r* = 0.38, *p* < 0.01.

## Discussion and Conclusion

The present study investigated if instructions on strategies for illustrated text reading that focused on illustration reading and text-illustration integration helped young readers overcome their deficiencies, and change reading processes, learning outcomes, and subjective perceptions of article difficulty and enjoyment, illustration enjoyment, and self-evaluation of learning. Overall, the results showed that reading strategy instructions promoted learning outcomes, changed reading processes, but did not affect subjective perceptions of the article in fourth-grade students.

### Reading Instructions Promote Learning Outcomes and Influence Reading Processes

Several results in this study confirm that reading strategy instructions promote learning outcomes and influence cognitive processes involved in reading illustrated science texts.

First, the instruction group outperformed the control group on the reading test, especially for illustration recognition and text-illustration integration questions. This replicates the previous research (Jian, 2018), and can be explained by dual-coding theory (Paivio, 1990), which suggests that readers who use and integrate textual and pictorial representations outperform readers who only use single representations to construct mental models of leaning materials. In this study, reading strategy instructions led to the greatest improvements in memorizing illustration information and integrating text and illustration representations.

Second, the instruction group spent more reading time on the article and illustration section than the control group. This indicates that individuals who have learned reading strategies are willing to devote more mental effort and time to reading for comprehension. They also knew that science illustrations are important (Pozzer and Roth, 2003; Slough et al., 2010), so need to be read carefully to decode information.

Third, the instruction group spent a greater proportion of reading time (27%) on illustrations (top and bottom) than the control group (16%). Interestingly, the instruction group’s proportion of reading time spent on illustrations was similar to the proportion observed in adult readers (26%; Jian, 2016), and more than that in a previous study (Hannus and Hyönä, 1999), which showed that students spent little time (about 6%) looking at illustrations in a biology article. This indicates that students in the instruction group learned the illustration reading strategy that the majority of young readers do not develop naturally at that age (Moore and Scevak, 1997; Hannus and Hyönä, 1999; Norman, 2012; Jian, 2016, 2018), and they directed attention to illustration sections because they recognized the importance of the science illustrations. In contrast, students in the control group demonstrated text-driven reading (Hannus and Hyönä, 1999), and spent significantly more of their reading time (84%) on the text than the instruction group. Additionally, as shown in Table 4, the proportion of total reading time for the two groups only differed significantly for paragraph 2, suggesting that participants in the instruction group were aware that paragraph 2 had important reading comprehension contents. Therefore, they needed to make more cognitive efforts to read those sentences.

Fourth, the instruction group made more saccades between the text and illustrations than the control group, especially between related text and illustration sections (paragraph 2 to top illustration, paragraph 3 to bottom illustration). This indicates that reading instructions changed learning processes, helping readers use multiple representations during reading, leading to better learning. This change in reading processes and learning outcomes can be explained by the cognitive theory of multimedia learning (Mayer, 2014) and integrated model of text and picture comprehension (Schnotz and Bannert, 2003; Schnotz et al., 2014).

### Reading Strategy Instructions Did Not Influence Subjective Perception of the Article

Reading instructions had no influence on subjective perceptions of the article. This may be because the reading instructions were brief (approximately 10–15 min), and not long enough to change metrics of reading attitude, such as article enjoyment and illustration enjoyment, of self-evaluation of learning. Although students who received reading strategy instructions learned better, they did not think that they did. Future research should measure teaching and learning time to examine the effect of instructions on subjective perception. Alternatively, it may be that the instructions only focused on cognitive strategies, which may not change affective attitudes. Future research should combine reading strategies with teaching activities to stimulate experiences, emotions, and prior knowledge that readers can associate with text content (Henschel et al., 2016).

### Subjective Perception of the Article Was Not Correlated With Reading Behavior and Reading Performance

There was no relationship between subjective perception of the article, reading behavior, and reading performance. That is, there were no relationships between article difficulty or enjoyment and reading time or reading comprehension test score, nor between illustration enjoyment and reading time. This is inconsistent with previous work showing that interest is related to engagement (Ainley, 2006; Unrau and Quirk, 2014). It may be that some participants felt the science article was very difficult and devoted a lot of time and mental effort to process it; however, some participants may have done the opposite, and given up sooner; therefore, these positive and negative correlations may have cancelled each other out.

### Empirical and Practical Contributions

Empirically, this study developed new instruction methods in multimedia learning and further examined their effect on learning processes in young readers. In most previous research, text-illustration integration was encouraged by designing signals on the reading materials (Scheiter and Eitel, 2015) or using eye-movement modeling examples (van Gog et al., 2009; Mason et al., 2015). Readers in these studies were middle-school or university students, and the conclusions are limited in their ability to explain facilitation effects in young readers. Visual literacy is a cognitive ability that develops late (Kress and van Leeuwen, 1996; Moore and Scevak, 1997), and young readers may not have sufficient ability or experience to decode illustration meaning (McTigue and Flowers, 2011), resulting in seeing but not understanding (de Koning et al., 2010). In addition, we extended explanations based on multiple learning theories (c.f., cognitive theory of multimedia learning, Mayer, 2014, and integrated model of text and picture comprehension, Schnotz and Bannert, 2003; Schnotz et al., 2014) to young readers. If young readers receive effective reading instructions, they can use textual and pictorial representations to construct better mental models during illustrated text reading.

Practically, this study can help elementary school teachers understand the processes used by young readers when reading illustrated texts and provide them with evidence-based instructions to teach science reading effectively. Clear instructions and demonstrations for three reading strategies are provided in the present study. It was inspiring to find that illustrated text reading strategy instructions changed learning processes and outcomes in young readers. If readers learn good reading strategies, they will acquire abundant scientific knowledge and concepts by themselves rather than relying on teacher instruction.

### Limitations and Future Directions

Some research limitations and future research directions are proposed. The effect of reading strategy instructions was stronger for illustration memorization and text-illustration integration than for text memorization and comprehension. It may be that the three reading strategies did not emphasize text comprehension that often improves via inference and prediction strategies. Future studies should investigate this assumption by combining the three reading strategies used here with other reading strategies (e.g., inference and prediction strategies) often used in pure text reading (Hansen and Pearson, 1983; McGee and Johnson, 2003; Hall, 2016). Furthermore, future studies may also consider eye-movement modeling example (EMME) to evaluate its effects compared to reading instructions, since previous research has demonstrated the effects of EMME only on middle-school students (Jarodzka et al., 2013). If EMME is effective for young readers, then elementary school teachers can choose either one or combine both methods, depending on the teaching situation or learners.

Moreover, most research investigating instruction effects on reading use quasi-experimental designs in the school environment, whereas the present study adopted an experimental paradigm to investigate the effect of reading instructions on learning processes and outcomes. Thus, the ecological validity of these results could be questioned because they were not obtained in a real classroom environment. However, it is difficult to consider external and internal validity in preliminary studies. In this case, an experimental design was adopted because studies on illustrated text reading strategies are rare, and I wanted to examine their effects in rigorously controlled conditions. After demonstrating the validity of these instruction methods, future research will progress to real classrooms. In addition, the reading instructions were brief. Although the experimenter used several methods (e.g., asking individuals to describe each reading strategy with examples in a practice article, viewing on-line eye movements on the practice article) to check that students learned the three reading strategies, the test only reflected that students learned these reading strategies in a short time, and could transfer them to a new science article. Future research should include a delayed reading condition and test to investigate if the acquired reading strategies still produce beneficial effects 1 or 2 weeks later.

Despite these limitations, there was a positive effect of reading instructions on reading processes and learning outcomes during illustrated text reading of material that described the structure and functions of a flower. This is a common topic in biology texts, and is often used in research on learning and instructions (Ainsworth and Loizou, 2003; Butcher, 2006; Scheiter and Eitel, 2015). Future research should expand the types of reading materials used to investigate if illustrated text reading strategies that emphasize illustration reading and text-illustration integration also foster learning outcomes for other science articles.

## Author Contributions

Y-CJ designed this study, reviewed the relevant literature, conducted the eye-movement experiment, analyzed data, and wrote this manuscript.

## Conflict of Interest Statement

The author declares that the research was conducted in the absence of any commercial or financial relationships that could be construed as a potential conflict of interest.
