# Supplementary material for: Reading Instructions Influence Cognitive Processes of Illustrated Text Reading Not Subject Perception: An Eye-Tracking Study
Source: Front Psychol. 2018 Nov 29;9:2263. doi: 10.3389/fpsyg.2018.02263 (PMC6281751; doi:10.3389/fpsyg.2018.02263)
Supplement: Supplementary file 1 [file Data_Sheet_1.pdf]

Appendix A. The reading self-efficacy questionnaire and the subjective perception questionnaire

The reading self-efficacy questionnaire

1. I usually do well in reading.
2. Reading is easy for me.
3. Reading is more difficult for me compared to most of my classmates.
4. I can read to learn more than most of my classmates.
5. If a book is very interesting, then even if it is difficult to read, I still have to read it.
6. It is hard for me to read a story with difficult words.
7. I feel reading is more difficult than other subjects.

The subjective perception questionnaire

1. Do you think the article is difficult?
2. Do you like the article?
3. Do the science illustrations in the article attract you? (Do you like to look at the science illustrations?)
4. How much did you learn from reading the article?

Appendix B *Examples of the reading test*

Recognition questions (multiple-choice items)

(Textual item) "Which one appeared in the article that you read?

(1) Petals are above the sepal.

(2) Petals are inside the sepal."

(Diagram item) "What is the component of the flower that the arrow is pointing to? (1) anther (2) thrum (3) style"

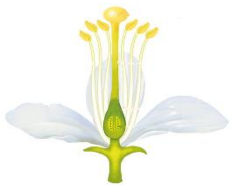

Comprehension questions (yes-or-no items)

(Textual item) "Does the ovary develop into the seed?"

(Diagram item) "Is the style thin and long?"

(Integral items) "Does the sepal have the function of protecting stamen and pistil, as well as attracting

insects for pollination?”
